# The role of low-level image features in the affective categorization of rapidly presented scenes

**DOI:** 10.1371/journal.pone.0215975

**Published:** 2019-05-01

**Authors:** L. Jack Rhodes, Matthew Ríos, Jacob Williams, Gonzalo Quiñones, Prahalada K. Rao, Vladimir Miskovic

**Affiliations:** 1 Department of Psychology, State University of New York at Binghamton, Binghamton, New York, United States of America; 2 Computer Science and Engineering, University of Nebraska, Lincoln, Nebraska, United States of America; 3 Mechanical and Materials Engineering, University of Nebraska, Lincoln, Nebraska, United States of America; University of Colorado Boulder, UNITED STATES

## Abstract

It remains unclear how the visual system is able to extract affective content from complex scenes even with extremely brief (< 100 millisecond) exposures. One possibility, suggested by findings in machine vision, is that low-level features such as unlocalized, two-dimensional (2-D) Fourier spectra can be diagnostic of scene content. To determine whether Fourier image amplitude carries any information about the affective quality of scenes, we first validated the existence of image category differences through a support vector machine (SVM) model that was able to discriminate our intact aversive and neutral images with ~ 70% accuracy using amplitude-only features as inputs. This model allowed us to confirm that scenes belonging to different affective categories could be mathematically distinguished on the basis of amplitude spectra alone. The next question is whether these same features are also exploited by the human visual system. Subsequently, we tested observers’ rapid classification of affective and neutral naturalistic scenes, presented briefly (~33.3 ms) and backward masked with synthetic textures. We tested categorization accuracy across three distinct experimental conditions, using: (i) original images, (ii) images having their amplitude spectra swapped within a single affective image category (e.g., an aversive image whose amplitude spectrum has been swapped with another aversive image) or (iii) images having their amplitude spectra swapped between affective categories (e.g., an aversive image containing the amplitude spectrum of a neutral image). Despite its discriminative potential, the human visual system does not seem to use Fourier amplitude differences as the chief strategy for affectively categorizing scenes at a glance. The contribution of image amplitude to affective categorization is largely dependent on interactions with the phase spectrum, although it is impossible to completely rule out a residual role for unlocalized 2-D amplitude measures.

## Introduction

Perceptual processing in the natural world is strongly influenced by motivational factors, allowing for adaptive behavioral routines in response to threats and opportunities in the environment [1–4]. Complex scenes can be affectively discriminated even with very rapid exposure times [5]. Enhanced brain physiological responses elicited by emotional, relative to neutral, scenes are detectable by ~200 ms using non-invasive recordings [6–8], although earlier latency modulations are apparent in intracranial studies [9–10]. Given how rapidly affectively salient information is extracted from these rich, visually cluttered stimuli, it remains to be understood how the human visual system accomplishes this feat [2].

One strategy that may enable fast recognition of image content is the use of low-level features, such as the distribution of contrast across spatial frequencies [11–15]. The visual system forms a rough, initial sketch of complex scenes based on such low-level sources of information, which can later be filled in with higher level processing. The affective Gestalt of scenes (i.e., whether they signal something aversive or neutral) is inseparable from the lower, physical aspects [16–17] and emerges only after these more basic semantic kinds of categorization have occurred [18–21].

**Fig 1:**
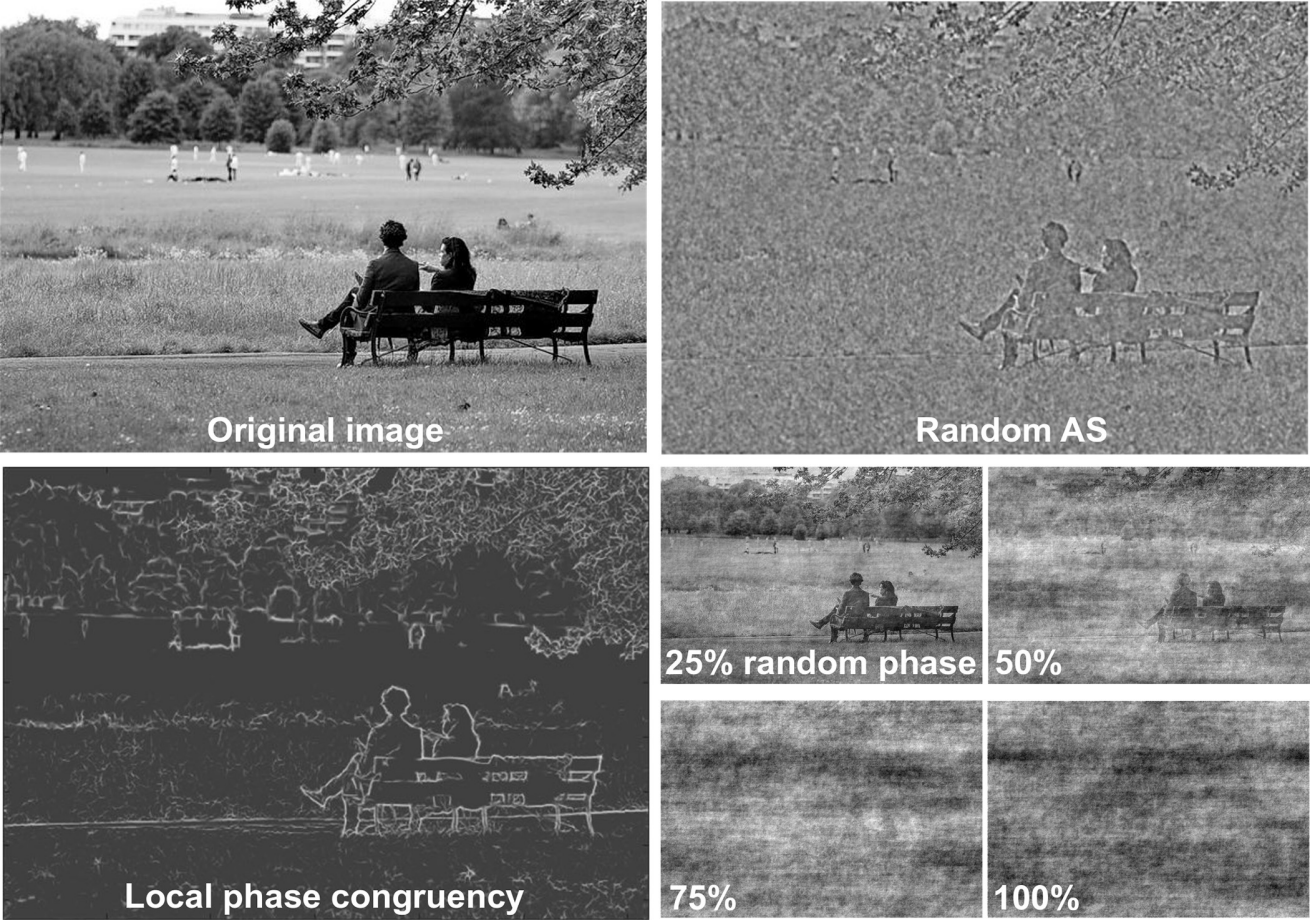
The upper row depicts an intact scene (*top left*) and the same scene with the phase spectrum left intact but with amplitude spectra wholly randomized (*top right*). The lower panel depicts the local phase congruency (LPC) map of the intact image (*bottom left*), where darker greys indicate relatively lower phase congruency and lighter grays indicate higher LPC, corresponding to local edge and corner detail. The bottom right panel illustrates variants of the original image with an unaltered amplitude spectrum, though with varying amounts of phase randomization. Note the degradation of edge and corner detail as phase randomization increases.

One candidate feature that might be used to guide the extraction of affective tone from a simple glance is the 2-D Fourier amplitude spectrum of images, which might be sufficient to capture basic statistical regularities in the luminance contrast of different types of real-world scenes [22]. A 2-D Fourier transform provides a way to represent an image as a complex, 2-D luminance waveform, consisting of a sum of sinusoidal waveforms with different spatial frequencies, amplitudes, orientations, and phases, from 0 to 2π [23]. The phase spectrum has long been known to be more important than the amplitude spectrum for parsing image semantics [24], as it carries the higher-order statistical relationships of pixel luminance values [25]. The phase spectrum of a scene conveys information about local and global scene features, such as the edge and corner locations (occurring where phase is congruent at neighboring spatial locations) [23, 26–27]. As illustrated in Fig 1, the phase spectrum indicates where, within a scene, different frequencies are aligned: phase values at nearby locations are highly similar, or congruent, where local edges and corners occur, and are more randomly distributed in areas in which these structural features are absent [26, 28–30]. The Fourier amplitude spectrum, on the other hand, is unlocalized insofar as it provides information about image contrast as a function of spatial frequency and orientation, independent of location in the spatial domain. Although the amplitude spectra representation of an image is relatively crude and contains no recognizable structure, there is intriguing evidence that it may be useful in generating an initial sketch or basic gist of natural scenes [31].

Computational models suggest that it is possible to obtain high levels of scene categorization accuracy on the basis of Fourier spectral amplitude information alone [31–32]. For human observers, the categorization of rapidly presented natural scenes is strongly primed by the statistical properties of Fourier amplitude spectra under different task designs [33–34], while performance tends to suffer somewhat, both in macaque monkeys and humans, when the Fourier amplitude spectra of images are equalized across semantic categories [35]. Amplitude spectra seem to be especially informative in the rapid classification of human faces, a stimulus class that is rich in biological significance [36–38]. Contrary to these findings, other studies have found that image amplitude spectra (unlike phase spectra) are not necessary or sufficient for the rapid categorization of global scenes and in animal detection tasks [12, 39–42].

It is presently unclear whether the low-level features present in 2-D Fourier amplitude spectra are used to guide the rapid extraction of emotion regularities from complex natural scenes. It is feasible that scenes depicting mutilation and injury, like faces, represent a special stimulus category with high biological significance, such that the human visual system capitalizes on information contained in unlocalized 2-D amplitude spectra for rapid recognition, even if such information is not obviously related to the semantic content of a scene. Although as a category, human faces are known to possess a very narrow range of spatial frequency characteristics useful for face identification [43–44], it remains unknown whether specific categories of complex affective scenes differ from neutral scenes in their spectral information. Delplanque et al. [45] have suggested that energetic differences among emotional and neutral scenes might be sufficient to support their discrimination, especially during an initial “quick-and-dirty” processing stage (e.g., [46]). In keeping with this possibility, several studies have reported interactions between affective scenery and energetic content in specific spatial frequency ranges in modulating brain responses for emotionally arousing content [46–48].

As an initial, proof-of-concept validation, we wished to determine whether Fourier image amplitude carries discriminative information about the affective quality of scenes. To test this possibility, we first designed and evaluated a support vector machine (SVM) classifier using amplitude-only image features as inputs. This model allowed us to answer whether, in principle, images belonging to different affective categories could be distinguished purely on the basis of their amplitude spectra. To the extent that amplitude spectra enable above-chance categorization of these stimuli, it becomes feasible that such features are exploited by the visual system when affectively labeling rapidly presented scenes.

Subsequently, we tested the performance of human observers on a task examining affective classification of affective and neutral scenes, presented briefly (~33.3 ms) and backward masked with synthetic textures. The categorization of naturalistic images under such a challenging set of spatio-temporal presentation constraints tentatively emphasizes the initial, feedforward sweep of activation through the visual system [49–50], while minimizing opportunities for recurrent processing [51]. We selected two affective sub-categories of aversive scenes, mutilation and disgust. The rationale for these narrow categories was that, first, we wished to avoid an overly broad selection of aversive images (e.g., scenes depicting sadness, which are often rated as lower in emotional arousal), and instead wanted to impose some restriction in the range of variability physical image properties. Second, previous evidence from intracranial recordings in the human amygdala [10] and fMRI investigation of the insula [52] suggest that these two sub-categories of aversive content recruit distinct neuronal populations, and may constitute ‘natural kinds’ of a sort.

In order to evaluate the specific contribution of Fourier amplitude spectra in affective categorization, we adapted an approach developed by Gaspar and Rousselet ([12]; see also [36]). More specifically, we tested behavioral accuracy across three distinct experimental conditions, using: (i) original images, (ii) images having their amplitude spectra swapped within a single affective image category (e.g., an aversive image whose amplitude spectrum has been swapped with another aversive image) or (iii) images having their amplitude spectra swapped between affective categories (e.g., an aversive image containing the amplitude spectrum of a neutral image). Exploiting this form of image manipulation allowed us to control for performance decrements arising due to the increased edge noise associated with perturbing amplitude-phase interactions following amplitude swapping. We thus expected higher performance for the intact images relative to those in both of the amplitude spectra swapped conditions. We also expected that if unlocalized 2-D Fourier amplitude spectra influence the affective categorization of scenes, then classification performance ought to be higher for images that have had their spectra swapped within, compared to between, affective image categories. On the other hand, if accuracy suffers equally in the two swapped relative to intact experimental conditions, then this would suggest that amplitude information alone plays no role in image categorization and that it only becomes relevant in the context of an interaction with the phase structure of natural scenes. To determine how and whether these low-level image manipulations impact evaluative responding to images, we also collected subjective valence and arousal ratings in a separate task.

## Method

### Participants

A total of 97 undergraduate students at SUNY Binghamton participated in all experimental procedures in exchange for partial course credit. Three participants were excluded from analyses for providing the same categorization responses on ≥ 90% of all trials and four participants were excluded for an excessive number of time-outs (> 15% of trials) during the rapid scene categorization task. The remaining 90 participants (68 female) had a mean age of 18.83 (S.D. = 1.45) years. All procedures were approved by the SUNY Binghamton Institutional Review Board.

### Stimuli

#### Image set

Each aversive image subcategory (mutilation and disgust) was paired with a unique subset of the neutral images (e.g., people at work, people in street scenes). Each of the four subcategories (two aversive, two neutral) contained 80 unique images, for a total of 320 images. Each of the subcategories contained a mixture of close-up and wide-angle images. Images were drawn largely from extant publicly available research datasets normed for valence and arousal, including the EmoPics [53], Geneva Affective Picture Database (GAPED, [54]), the International Affective Picture System (IAPS, [55]), the Nencki Affective Picture System (NAPS, [56]), the Military Affective Picture System (MAPS, [57]), the Open Affective Standardized Image Set (OASIS, [58]) datasets, and were supplemented with pictures from Google image searches. A full list of images used in this study is available online at osf.io/5ztvf.

All images were resized to 1024 x 768 pixels, converted to greyscale to avoid introducing any interactions with specific color channels, and matched for luminance and contrast using the *lumMatch* function from the SHINE toolbox [59] as implemented in Matlab. Images, presented centrally, subtended a visual angle of 14.13 x 10.69 degrees at a 114 cm viewing distance on an Asus VN247 24-inch monitor (1920 x 1080 pixels, refresh rate = 60 Hz, response time ~ 1 ms).

#### Amplitude swapping

Images in our identity swapped (ID) condition were constructed through pairwise swapping of amplitude and phase spectra from two unique images within the same category (e.g. amplitude from one mutilation image matched with phase from another mutilation image). In a first step, both source images were Fourier transformed and the amplitude spectra of image A was then paired with the phase from image B and the amplitude spectra of image B paired with the phase of image A. In a final step, the complex Fourier coefficients were subjected to an inverse Fourier transform. This process was performed separately for all image categories. Our between-category swapped (BTW) images were constructed through an identical process, except for the critical difference that the amplitude swapping occurred between different image categories (e.g. amplitude of a mutilation image and phase of a neutral image). No amplitude swapping occurred in the intact condition.

**Fig 2:**
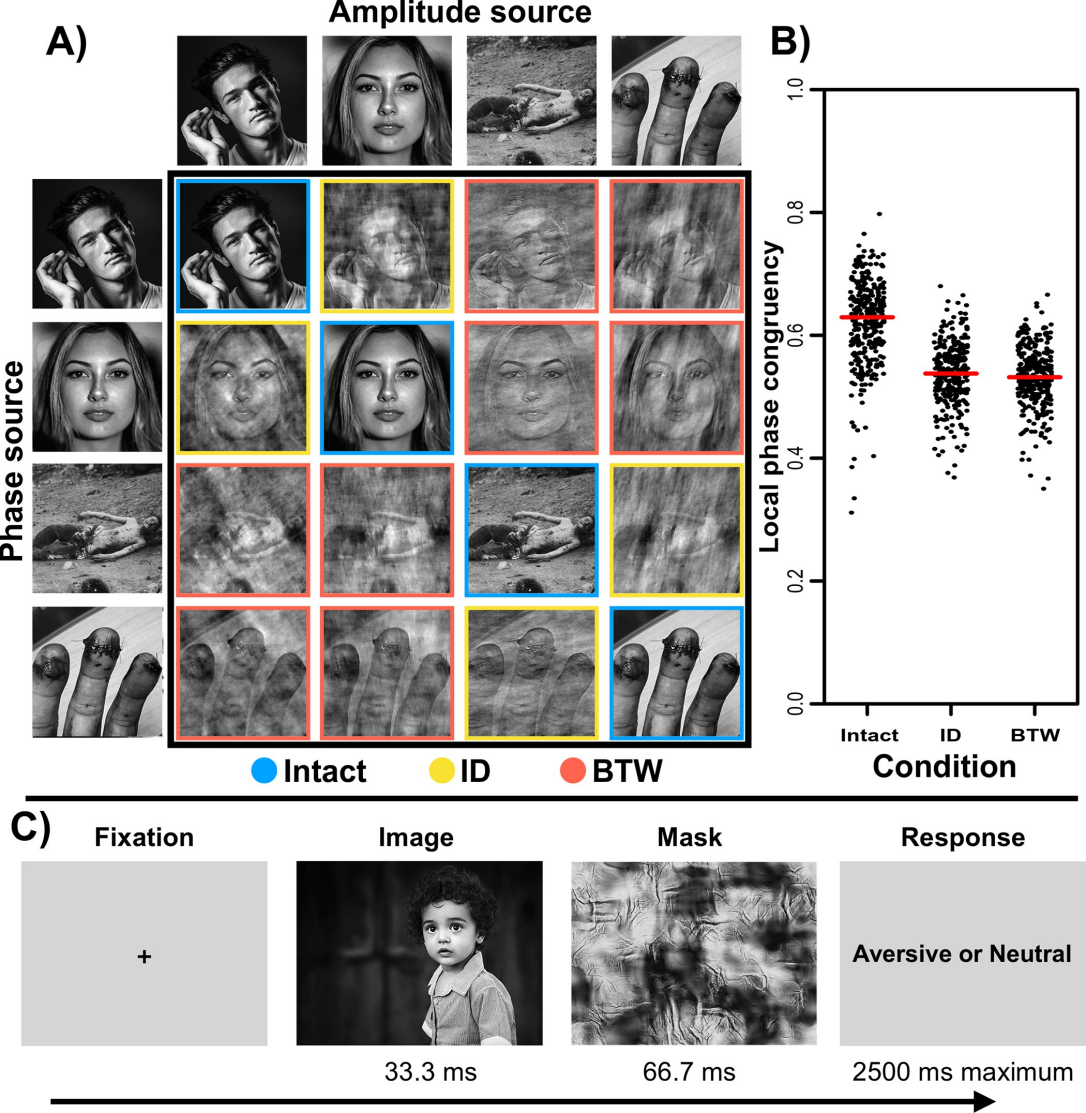
Panel A illustrates sample intact images alongside the distinct image manipulations, involving within (ID) and between (BTW) semantic category swapping of 2-D amplitude spectra. Panel B illustrates the local phase congruency (average of top 10 LPC values) for images in the intact, ID, and BTW conditions with the median for each group indicated by the red bar. Panel C illustrates the sequence for one rapid categorization trial. Note that the image exemplars depicted here are shown for illustrative purposes only and were not ones used in the actual experiment.

For analysis purposes, ground truth affective category labels for each image are based on the phase, not amplitude spectra, of that image. Fig 2A provides a schematic illustration of the image manipulation operations. Each participant was assigned to one of these three (intact, ID, BTW) image conditions.

Amplitude swapping introduces a considerable amount of edge noise, owing to a disturbance of interactions between image amplitude and phase, resulting in a ‘cloudy’ visual effect. To ensure that classification accuracy for the images used in the ID and BTW experimental conditions did not vary as a function of edge noise, we quantified image local phase congruency (LPC) measures using Kovesi’s [26, 29–30] *phasecong3* algorithm. As illustrated in Fig 2B, mean LPC (averaged across the 10 most salient locations in each image) differed significantly between the intact and ID and between the intact and BTW conditions, [*t*(638) = 17.50, *p* = 2.73 x 10^−56^] and [*t*(638) = 18.62, *p* = 3.93 x 10^−62^], respectively. Critically, LPC values did not differ between ID and BTW conditions (*p* = .20).

#### Texture masking

Each image was paired with a unique mask generated using the Portilla and Simoncelli [60] parametric texture model as implemented in Matlab. Previous work has demonstrated the effectiveness of these masks in the context of rapid scene categorization [61–62]. For each image, model inputs included the source image and an equally sized white noise matrix. This model generates a texture based on the statistics of input adjacent spatial scales, orientations, and locations (here set to 4, 4, and 7, respectively, with 25 iterations). Model output provided a synthetic texture mask unrecognizable as the original image, while containing many of the original image’s global higher order features. Masks were generated after creation of the luminance-normalized intact, ID, and BTW images (i.e., each mask had three variants, one for each amplitude spectrum–phase condition). On every trial, the mask presented was that generated from the target stimulus. We illustrate a representative image with its paired mask in Fig 2C.

### Machine learning

**Fig 3:**
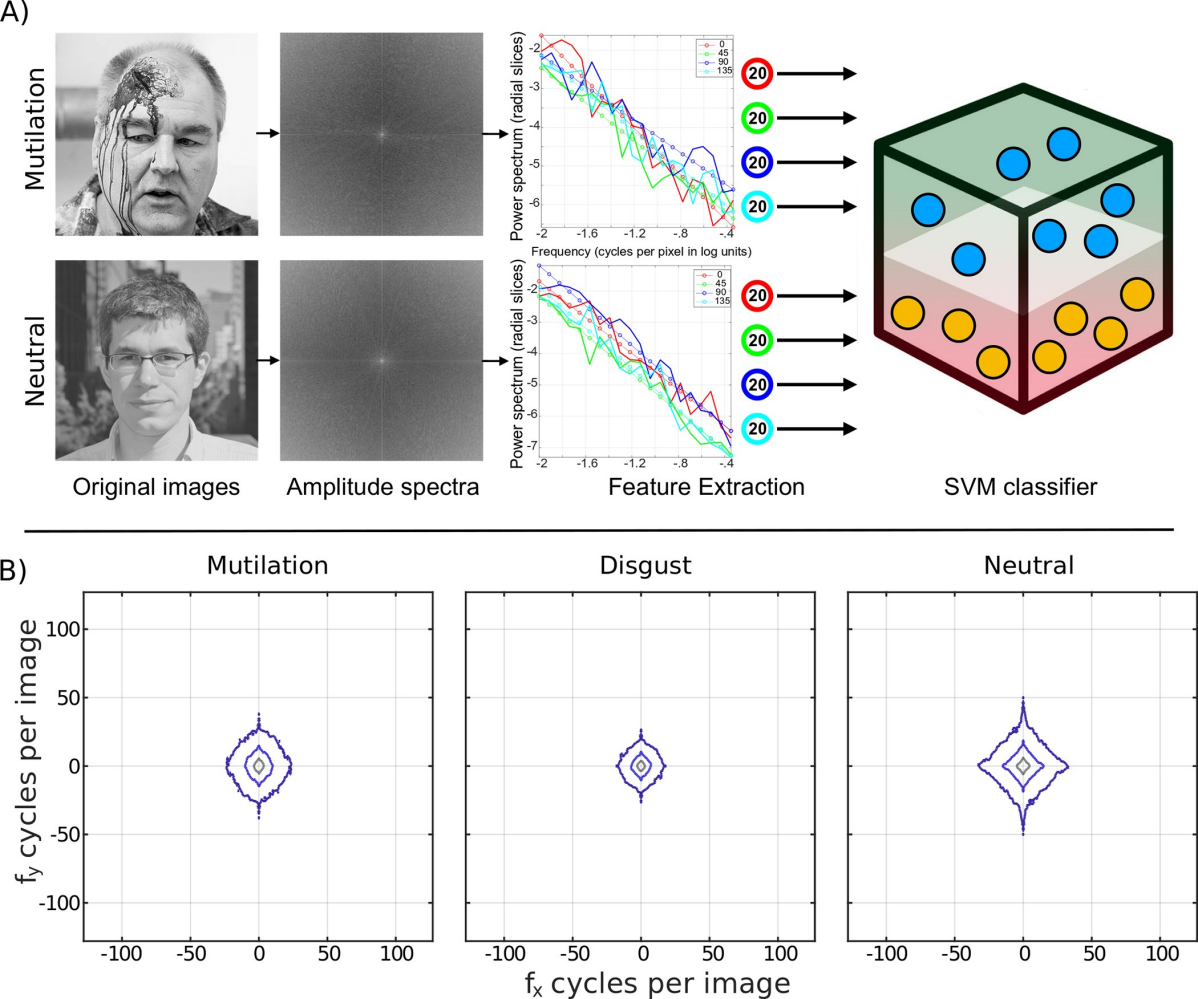
The upper panel illustrates the support vector machine (SVM) classification sequence for a representative mutilation and paired neutral image. First, one half of the original images were subjected to a two-dimensional fast Fourier transformation (2-D FFT). Phase spectra were discarded and the SVM was trained with 80 amplitude-only features per image (20 spatial frequencies, four orientations). After training, the SVM was tested on the remaining half of the images. The lower panel illustrates the averaged spectral contour plots of our intact mutilation, disgust, and neutral images. The inner contour represents 60% and the outer contours 80 and 90%, respectively, of image energy.

SVM is a supervised learning algorithm used in classification tasks. It is a binary classifier that maximizes the margin of the hyperplane separating data from the two classes in a projected space using the kernel trick [63]. It is effective in tasks with low data availability. The *Scikit-learn* toolbox [64], as implemented in Python, was used for classification and in determining cross-validation splits. Eighty features per image were extracted, containing only amplitude information, following Crouzet and Thorpe [36]. Briefly, images were first resized to 256 x 256 and windowed with a Hamming function to reduce boundary artifacts. Subsequently, a 2-D FFT was computed for each image, and four separate radial sections of the power spectrum were extracted into 45° orientation bins covering the two major cardinal (horizontal and vertical) and oblique orientations. Finally, we selected 20 points along a radial axis for each orientation bin, so that each image was summarized by a total of 80 features (see Fig 3 for a schematic). All features were extracted using the Natural Image Statistical Toolbox [65] as implemented in Matlab.

Classification was performed over two splits of the data: (i) mutilation versus neutral, and (ii) disgust versus neutral. Grid search was performed over the C and *γ* hyperparameters of a Gaussian SVM in order to determine an appropriate model. The C parameter acts as a regularizer on the objective of the SVM, determining the tradeoff between classification accuracy and margin width according to the loss function:

$$\min{\left|w\right|}^{2}+C\sum \xi$$

where *w* is the width of the margin and ∑*ξ* is the classification error. A higher *C* value will encourage higher training accuracy, at the potential cost of model generalizability. The *γ* parameter controls the shape of the Radial Basis Function used in the Gaussian SVM, with large *γ* corresponding to lower variance models and vice versa, given the formula:

$$K(x{,x}^{\prime })=exp(-\gamma {\left\|x-x^{\prime }\right\|}^{2})$$

For each of the two class problems, mutilation versus neutral and disgust versus neutral, repeated *k-*fold cross-validation was performed to evaluate each model in the grid search. A total of 8,000 classifications were performed over 50 replications of 10-fold cross-validation of the 160 images (80 per class) for each hyperparameter combination in the grid search. To explain further, the images were randomly split into 10 groups of 16 images each, 8 from each class, over which cross-validation was performed. This process was then repeated 50 times. This repetition of the *k*-fold procedure allowed for a lower variance estimate of performance, while maintaining the low bias inherent to *k-*fold cross-validation [66]. This is especially important in our task since, due to the limited availability and high dimensionality of the data, maintaining a completely independent test set during hyperparameter search is impractical. That is, the cost to model stability of further subdividing the data was determined to outweigh the cost of bias from hyperparameter selection without a fully independent test set. To further alleviate the bias induced by the lack of an independent test set, a full listing of the grid search results is available on OSF. A Gaussian SVM with a C parameter of 1 and *γ* parameter of 0.01 was found to yield strong performance in both tasks, and was chosen for the final evaluation. A separate application of the repeated *k-*fold procedure was performed to determine the final results.

### Procedure

Following the completion of a written consent, participants were screened for 20/20 vision using a Snellen eye chart. We used a between-subjects design as a relative dearth of unpleasant, particularly disgust, image content precluded a within-subjects design as this would require image repetition and would introduce the confound of prior experience. Participants were randomly assigned to one of the three experimental conditions and seated at a computer station, with a 114 cm viewing distance maintained through the use of a chin rest with an integrated forehead restraint. The computer monitor was the only source of room illumination (34.50 cd/m^2^). After review of verbal and on-screen instructions, participants completed a 15-trial practice session with stimuli not used in the experiment proper. Participants next completed the rapid scene categorization task, the valence and arousal rating task, and were thanked, debriefed, and released.

#### Rapid scene categorization task

Affective categorization consisted of a two-alternative forced choice (2AFC) task programmed in PsychoPy [67]. The task was programmed to display images and masks for two and four frames, respectively (versus a time-based 33 or 67 ms), to help mitigate against potential dropped-frame issues. Mutilation and disgust images, together with their paired neutral images were blocked such that one block contained 80 mutilation and 80 neutral images and the other block contained 80 disgust and a separate 80 neutral images. Block order was counterbalanced between subjects. Image presentation order was randomized for the 160 images in each block. Following a fixation cross (500 ms), each image was presented for 2 refresh frames (33.3 ms) and immediately backwards masked for 4 refresh frames (66.7 ms), resulting in a 1:2 target-to-mask ratio. A response prompt was then presented *(“Aversive = Left; Neutral = Right”*), and participants clicked one mouse button or the other to indicate their affective categorization decision (response options were counterbalanced between subjects). The response timer started at target image onset and the response window timed out 2.6 sec after target image onset if no response was given. Time-outs were coded as null responses and discarded prior to analyses, alongside trials with reaction times of less than 200 ms. Fig 2C illustrates the rapid scene categorization trial sequence.

### Valence / arousal ratings task

To assay image valence and arousal, participants rated each image on both affective dimensions after completing the rapid scene categorization task. Each image was presented for 33.3 ms (unmasked), immediately followed by a 9-point sliding valence scale based on the IAPS self-assessment manikin rating system (SAM, [55]), with the anchors *‘Very happy’* and *‘Very unhappy’*, and with a center point labeled *‘Neutral’*. Immediately after responding, participants rated image arousal on a 9-point sliding scale with the anchors *‘Intense’* and *‘Calm’*. Each participant rated all 320 images. Block and image presentation order were randomized. Intertrial intervals for both the rapid scene categorization and rating tasks were 0.5 to 1 sec, randomized. We used unmasked images for the affective ratings portion of the experiment as pilot work in our laboratory indicated that participants reported poor ability to report subjectively experienced affective tone when asked to rate the valence and arousal of immediately masked images. Moreover, previous work suggests that backward masking prevents the emergence of a SAM-based affective state space for image exposures less than ~ 80 ms [5].

### Statistical analyses

To measure differences in affective categorization accuracy across the three image conditions we calculated the Signal Detection Theory (e.g. [68]) sensitivity statistic *d* prime (*d’*) for each participant. Hit rates were quantified as the proportion of correct categorizations for aversive images and false alarms quantified as the proportion of neutral images categorized as aversive (computed separately for the mutilation and disgust blocks). *D* prime values for each participant, by image condition, were subjected to Kruskal-Wallis non-parametric testing with 5000 between-subject permutations. Participant image valence ratings were assessed with mixed model ANOVAs with valence ratings as the dependent variable, image condition (intact, ID, BTW) as a between-subjects factor, and ground truth image category (based on phase spectrum) as a within-subjects factor. Arousal ratings were assessed by the same model after replacing valence with arousal as the dependent variable. All ANOVA models were evaluated using Type III Sums of Squares. Greenhouse-Geisser corrections were applied where Mauchly’s tests indicated violations of the sphericity assumption. All parametric t-test multiple comparisons reported used Bonferroni correction of *p* values.

## Results

### SVM classification of affective and neutral scenes

To determine whether Fourier amplitude, independent of phase spectra, contains sufficient information to differentiate among scenes with distinct affective tone, we first considered the results from the SVM classifier. Only the results from the final hyperparameters as described in the methods section are presented here. However, the full grid search results available on OSF show that a broad set of C and *γ* hyperparameters were able to achieve similar performance.

As indicated in Table 1A, intact mutilation images were distinguished from neutral images with an accuracy of 74.95%. Neutral images were more likely to be misclassified as mutilation scenes compared to the converse, i.e., mutilation images being misclassified as neutral. In a similar vein, as shown in Table 1B, intact disgust images were distinguished from neutral ones with an accuracy of 70.36%. Neutral and disgust images were misclassified at similar rates (~30%), indicating that the approach is not biased to either case.

**Table 1:** (A) Intact mutilation images were distinguished from neutral images with 74.95% accuracy. Mutilation and neutral images were misclassified at similar rates (~30%), indicating that the approach is not biased to either case. (B) Intact disgust images were distinguished from neutral images with an accuracy of 70.36%.

**A. Confusion matrix for intact mutilation vs. neutral images**		Predicted	
		Neutral	Mutilation
Ground Truth (Known Labels)	Neutral (4000)	2793	1207
	Mutilation (4000)	797	3203
**B. Confusion matrix for intact disgust vs. neutral images**		Predicted	
		Neutral	Disgust
Ground Truth (Known Labels)	Neutral (4000)	2748	1252
	Disgust (4000)	1119	2881

The SVM classifier findings provide a proof-of-concept demonstration that Fourier amplitude information alone can, in principle, be used to achieve above chance affective discrimination of complex scenes. To find out whether this bias is exploited by humans to aid classification accuracy during rapid affective scene exposure, we next turned to considering the results of the 2AFC categorization task.

### Human rapid categorization performance

#### d’ index

**Fig 4:**
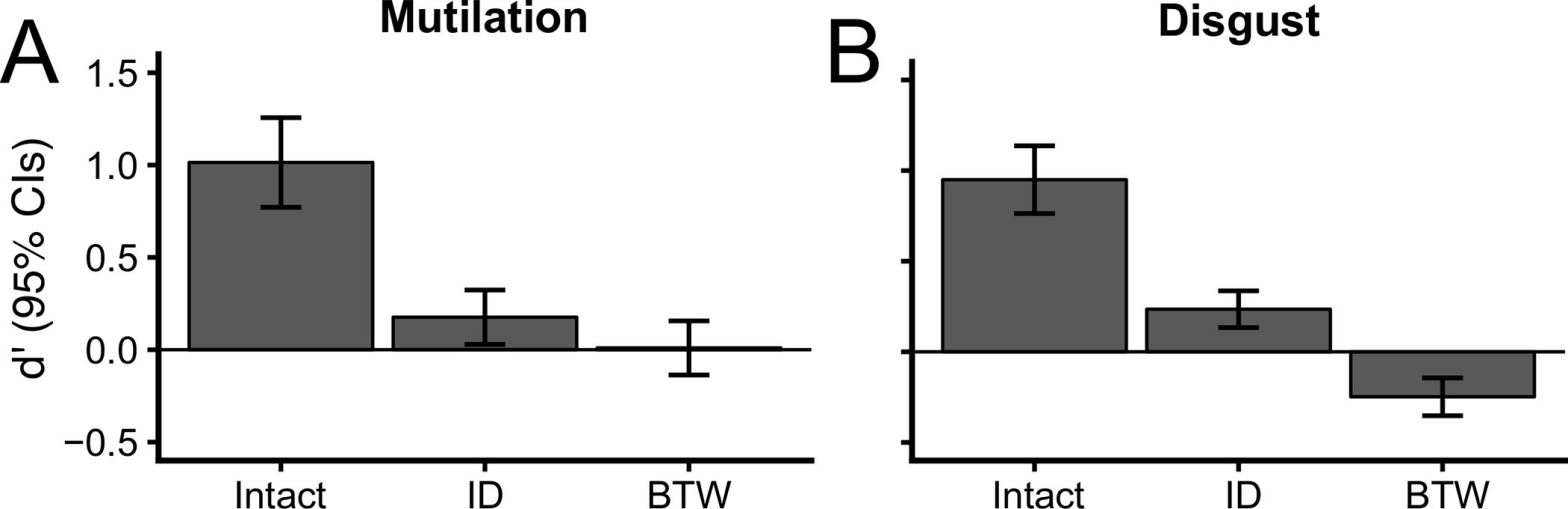
Panels A and B depict perceptual sensitivity (*d’*) values in each of the image conditions, for the mutilation and disgust blocks. Horizontal lines indicate chance performance. Error bars are 95% confidence intervals.

Results of the Kruskal-Wallis tests revealed differences in sensitivity for the three image conditions, in both mutilation versus neutral, *H*(2,87) = 38.24, *p* < 2.2 x 10^−16^, and disgust versus neutral categorizations, *H*(2,87) = 59.85, *p* < 2.2 x 10^−16^. As illustrated for the mutilation versus neutral categorization (Fig 4A), pairwise comparisons using Wilcoxon-Mann-Whitney tests demonstrated higher *d’* in the intact conditioned compared to both of the amplitude swapped (ID and BTW) image sets (*Z* = -4.98, *p*_perm_ = < 2.2 x 10^−16^ and *Z* = -5.53, *p*_perm_ = < 2.2 x 10^−16,^ respectively). *d’* did not differ significantly between the ID and BTW conditions, *Z* = -1.37, *p*_perm_ = 0.18. Subsequent one-sample *t*-tests versus zero (chance) indicated that d’ values for mutilation content was significantly above chance for the intact and ID images, *p*_perm_ = 2.09 x 10^−9^ and .02, respectively. By contrast, *d*’ values for BTW images did not differ from chance, *p*_perm_ = .89.

Results from the disgust versus neutral 2AFC categorization are shown in Fig 4B. We found higher *d’* values for intact relative to the ID and BTW images (*Z* = -5.14, *p*_perm_ = < 2.2 x 10^−16^ and *Z* = -6.45, *p*_perm_ = < 2.2 x 10^−16^). Additionally, categorization based on BTW images resulted in a lower *d’* than performance in the ID swapped condition (*Z* = 5.12, *p*_perm_ < 2.2 x 10^−16^). Separate comparisons against chance revealed that d’ values were significantly above chance for intact and ID image conditions (*p*_perm_ = 2.71 x 10^−11^ and 5.14 x 10^−5^, respectively). The images with between category amplitude swapping (BTW) produced below chance performance, *p*_perm_ = 3.71 x 10^−5^.

#### Valence and arousal ratings

**Fig 5:**
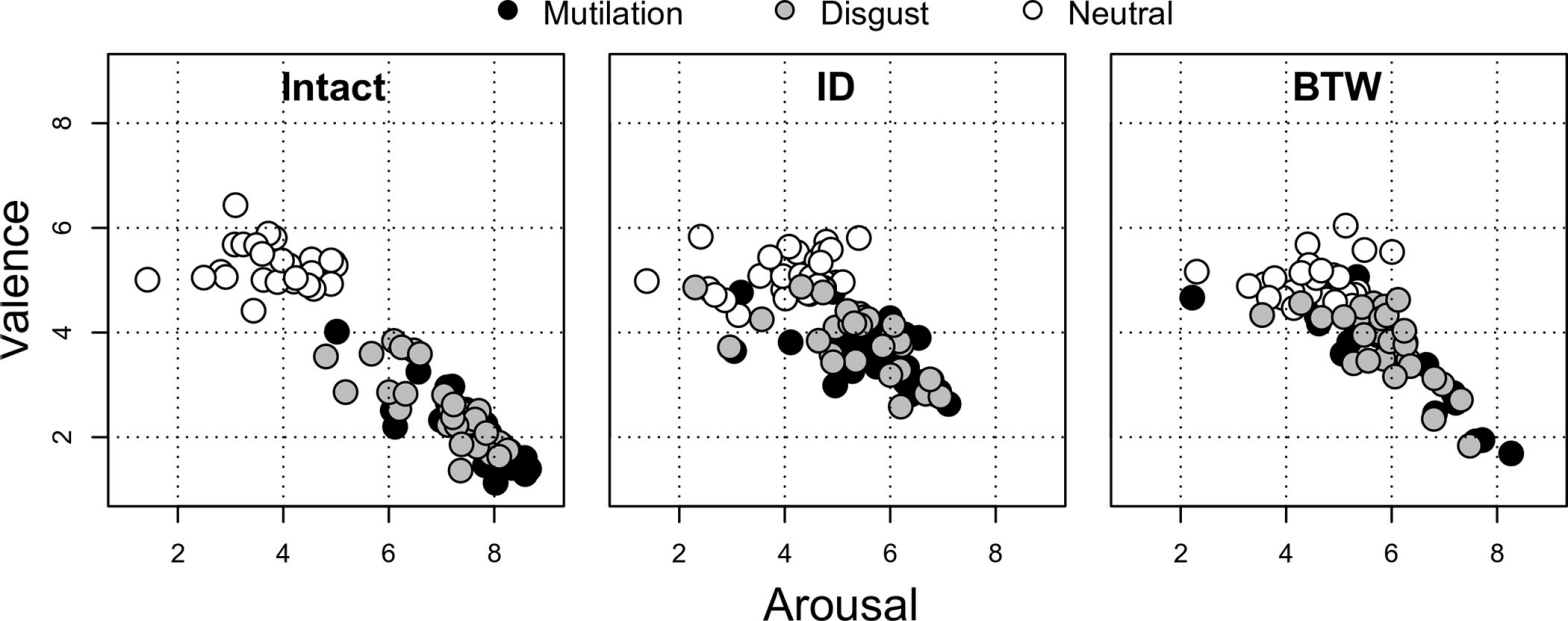
Neutral and aversive image ratings plotted in a 2-D affective space with valence and arousal each rated between 1 and 9 (with a valence rating of 5.0 being perfectly neutral). Each dot represents the mean rating for one participant for the indicated affective category.

Fig 5 depicts the structure of affective space, by participant, for images rated across the three different experimental conditions.

#### Valence dimension

Our ANOVA model revealed significant main effects of image condition, *F*(2,87) = 37.35, *p* = 1.95 x 10^−12^, η^2^_p_ = .46, and image type (mutilation, disgust, neutral), *F*(2,174) = 428.76, Greenhouse-Geisser corrected *p* = 5.71 x 10^−54^, η^2^_p_ = .83. These effects were qualified by an interaction of image condition and image type, *F*(4,174) = 40.90, Greenhouse-Geisser corrected *p* = 1.84 x 10^−19^, η^2^_p_ = .49. In order to break down this two-way interaction, we next conducted three separate ANOVAs examining the effect of the between-subjects factor of image condition for mutilation, disgust and neutral scenery. The main effect of image condition for mutilation scenes was significant, *F*(2,87) = 50.41, *p* = 2.90 x 10^−15^, η^2^_p_ = .54. As expected, intact mutilation scenes (*M* = 2.08, *S*.*D*. = .65) were rated as more aversive than those in the ID (*M* = 3.64, *S*.*D*. = .61) and BTW conditions, (*M* = 3.79, *S*.*D*. = .90), *p*s = 4.2 x 10^−12^ and 9.9 x 10^−14^, respectively. Similarly, the main effect of image condition for disgust imagery was significant, *F*(2,87) = 37.09, *p* = 2.25 x 10^−12^, η^2^_p_ = .46. Intact disgust images (*M* = 2.53, *S*.*D*. = .69) were rated as more aversive than those with ID (*M* = 3.82, *S*.*D*. = .60) and BTW swapping, (*M* = 3.80, *S*.*D*. = .70), *p*s = 1.4 x 10^−10^ and 2.5 x 10^−10^, respectively. The valence of neutral images also differed as a function of image condition, *F*(2,87) = 3.42, *p* = .03, η^2^_p_ = .07, with BTW swapped images rated as lower in valence (*M* = 4.97, *S*.*D*. = .38) compared to intact scenes (*M* = 5.24, *S*.*D*. = .40).

Despite the obvious warping of the affective space introduced by our image manipulation operations (as shown in Fig 5), mutilation and disgust images continued to be rated as more aversive than neutral ones for both the ID and BTW swapped amplitude spectra images. Within both of the AS-swapped conditions, mutilation and disgust images were still rated as more unpleasant than neutral images (ID: Cohen’s *d*s = 2.65 and 2.53; BTW: *d*s = 1.71 and 2.08, respectively). There were no differences in valence between ID vs. BTW swapped mutilation or disgust images (all *p*s > 0.45).

#### Arousal dimension

In terms of the rated arousal, there were main effects of image condition, *F*(2,87) = 16.26, *p* = 1.00 x 10^−6^, η^2^_p_ = .27, and image type, *F*(2,174) = 311.20, Greenhouse-Geisser corrected *p* = 6.26 x 10^−45^, η^2^_p_ = .78. Similar to the valence findings, these main effects were qualified by an interaction of image condition and image type, *F*(4,174) = 37.24, Greenhouse-Geisser corrected *p* = 1.15 x 10^−17^, η^2^_p_ = .46. We again conducted separate ANOVAs examining the effect of between-subjects AS condition for each of the image categories, observing a significant main effect of AS condition for mutilation imagery, *F*(2,87) = 34.48, *p* = 9.41 x 10^−12^, η^2^_p_ = .44. As expected, mutilation scenes in the intact condition (*M* = 7.59, *S*.*D*. = .83) were rated as more arousing than in the ID (*M* = 5.62, *S*.*D*. = 1.02) and BTW conditions (*M* = 5.79, *S*.*D*. = 1.18), *p*s = 1.6 x 10^−10^ and 3.1 x 10^−9^, respectively. Highlighting the effect of AS condition across aversive categories, our model revealed a significant main effect of AS condition for disgust imagery, *F*(2,87) = 23.29, *p* = 7.95 x 10^−9^, η^2^_p_ = .35. Participants rated disgust images in the intact condition (*M* = 7.01, *S*.*D*. = .87) as more arousing than in the ID (*M* = 5.41, *S*.*D*. = 1.09) and BTW conditions (*M* = 5.86, *S*.*D*. = .83), *p*s = 8.6 x 10^−9^ and 2.3 x 10^−5^, respectively. Finally, the significant main effect of AS condition for neutral imagery, *F*(2,87) = 4.81, *p* = .01, η^2^_p_ = .10, together with follow-up analyses reveals that intact neutral scenes (*M* = 3.92, *S*.*D*. = .85) were rated as significantly less arousing than in the BTW (*M* = 4.58, *S*.*D*. = .77) but not the ID (*M* = 4.09, *S*.*D*. = .93) condition, *p*s = .01 and > .99, respectively. Intact mutilation imagery was rated as significantly more arousing than intact disgust and intact neutral imagery, Cohen’s *d*s = .67 and 4.36, respectively. Intact disgust images were rated as more arousing than intact neutral images, *d* = 3.58.

In a similar vein to the valence dimension, image arousal ratings in the amplitude spectra-swapped conditions differed as a function of image category. Observers in the ID condition continued to rate mutilation and disgust images as more arousing than neutral images, Cohen’s *d*s = 1.57 and 1.31, respectively, with the same pattern for observers in the BTW condition, *d*s = 1.22 and 1.61, respectively. Arousal ratings did not differ between mutilation or disgust images in the ID vs. BTW categories (all *p*s > 0.55).

## Discussion

We examined the utility of 2-D Fourier amplitude spectra in guiding the affective categorization of rapidly presented natural scenes. In a first step, we validated the existence of Fourier amplitude-based category differences through SVM classification based on amplitude-only features as inputs. The classification accuracy for pairwise comparisons of mutilation/neutral and disgust/neutral images was ascertained to be in the 70 to 75% range, well above chance. Subsequent findings from human observers who performed a rapid scene categorization task with backward masking indicated that AS information alone contributes only minimally to categorization performance. We observed a marked deterioration in categorization accuracy for both of the amplitude swapped image manipulations relative to intact scenes. Overall, these findings are consistent with evidence from animal classification tasks (e.g., [12, 42]) that low-level information provided by unlocalized 2-D Fourier amplitude spectra is not sufficient to enable high accuracy performance. To the extent that image amplitude contributes diagnostic information for categorizing scenes at a glance, it appears to be almost entirely dependent upon interactions with the image phase spectrum, which is generally viewed as conveying higher-level visual features (see also [69]).

To date, the most convincing evidence in favor of Fourier amplitude aiding categorization is for face stimuli, which may be biologically special, and during *ultra*-rapid perceptual decisions [36–37]. For more complex, natural scenes, such as those examined here, altering the interaction of amplitude and phase appears to have a destructive influence on image primitives, including edges and corners that are required to support rapid visual discrimination [12]. This destructive influence is captured by the measure of local phase congruency [26, 29–30], which is co-determined by image amplitude and phase, and which was equally reduced in both of our amplitude swapped image conditions (see Fig 2B).

Finally, we also examined higher-order evaluative reactions to different types of image manipulations by collecting SAM-based valence and arousal ratings in a separate task involving unmasked presentations. Here, we found pronounced distortion and compression along both dimensions of affective responding. Relative to intact imagery, both kinds of amplitude swapping operations decreased valence and arousal discriminations. Residual differences in valence and arousal between the separate image categories persisted even for swapped images, but crucially there was no reliable difference in the structure of affective space between the ID and BTW swapped conditions. This provides converging evidence that amplitude spectra alone are insufficient to determine the affective tone of visual images.

There are several limitations to the present work. First, we examined categorization performance for a relatively narrow band of all possible unpleasant content and we did not consider pleasant emotional scenes. Second, it remains possible that amplitude spectra play a stronger role under a different set of viewing conditions or during ultra-rapid saccadic decisions. Another limitation of the present study is the mixed use of close up through relatively wide-angle scenes. While a qualitative attempt was made by us to balance the use of wide angle and close up images across the distinct semantic categories, future work might use a stricter selection of perspective or a more thorough matching of image perspectives within and across categories.

### Conclusion

While our SVM results demonstrate that there is, in principle, sufficient information contained in low-level (Fourier amplitude) features to discriminate distinct affective image categories, there is little evidence that this is a main strategic solution for human observers determining the affective tone of briefly presented natural images. Based on the present results it appears that amplitude spectra alone are of relatively little use in extracting affective content from briefly presented scenes without additional higher-level information contributed by the phase spectrum. However, based on our results, it is also impossible to entirely discount a role for unlocalized 2-D Fourier amplitude features. We found that within-category amplitude swapped images seemed to benefit from a slight advantage in categorization accuracy relative to between-category amplitude swapping, suggesting that there is some, residual information conveyed by image contrast as a function of spatial frequency and orientation.
